# Einsamkeit als Gesundheitsrisiko: Eine narrative Übersichtsarbeit

**DOI:** 10.1007/s00103-024-03939-w

**Published:** 2024-08-08

**Authors:** Susanne Buecker, Anne Neuber

**Affiliations:** Fakultät für Gesundheit, Department für Psychologie und Psychotherapie, Universität Witten/Herdecke, Alfred-Herrhausen-Straße 50, 58455 Witten, Nordrhein-Westfalen Deutschland

**Keywords:** Einsamkeit, Psychische Gesundheit, Körperliche Gesundheit, Mortalität, Morbidität, Loneliness, Mental health, Physical health, Mortality, Morbidity

## Abstract

Einsamkeit wird zunehmend als bedeutendes Gesundheitsrisiko anerkannt. Diese Übersichtsarbeit fasst den aktuellen Stand der Forschung zusammen, indem sie die Auswirkungen von Einsamkeit auf die psychische und körperliche Gesundheit beleuchtet. Die Ergebnisse zeigen, dass Einsamkeit sowohl für die psychische als auch für die körperliche Gesundheit ein Risiko darstellt. Bisherige Übersichtsarbeiten zu den Auswirkungen auf die psychische Gesundheit zeigen, dass Einsamkeit insbesondere den Beginn einer Depression, einer sozialen Phobie und den Abbau kognitiver Funktionen vorhersagt. Außerdem kann Einsamkeit die Erholung von psychischen Störungen im Allgemeinen erschweren, was unter anderem auf den bidirektionalen Zusammenhang zwischen Einsamkeit und psychischer Gesundheit zurückgeführt werden kann. In Bezug auf körperliche Erkrankungen wurden bisher vor allem Herz-Kreislauf-Erkrankungen als Folge von Einsamkeit untersucht. Einsamkeit sagt das Auftreten von Herz-Kreislauf-Erkrankungen vorher und kann die Genesung von diesen erschweren. Übersichtsarbeiten zeigen zudem, dass Einsamkeit die Wahrscheinlichkeit für Suizidalität und Mortalität erhöht. Es wird jedoch auch auf den Mangel an Längsschnittstudien hingewiesen, der es erschwert, die kausale Wirkrichtung von Einsamkeit auf spätere Gesundheitseinschränkungen zu untersuchen. Die in dieser Übersichtsarbeit aufgezeigten gesundheitlichen Folgen von Einsamkeit, die teils irreversibel sind und Leiden sowie Beeinträchtigung verursachen können, unterstreichen die Bedeutung von Interventionen zur Reduzierung von Einsamkeit als präventive Maßnahme gegen sowohl psychische als auch körperliche Erkrankungen.

## Abstract (en)

Loneliness is increasingly recognized as a significant health risk. This review provides an overview of current research on the impact of loneliness on mental and physical health. The findings indicate that loneliness is a significant risk factor of both mental and physical health. Previous reviews focusing on mental health effects reveal that loneliness is associated with the onset of depression, social anxiety, and cognitive decline. Furthermore, loneliness can impede recovery from mental disorders in general, which can be partly attributed to the bidirectional interplay between loneliness and mental health. Regarding physical health, consequences of loneliness have been mostly studied in relation to cardiovascular diseases. Loneliness predicts the onset of cardiovascular diseases and impedes recovery from them. Reviews also highlight that loneliness enhances the likelihood of suicidality and mortality. However, this article also points out the shortage of longitudinal studies, complicating the investigation into how loneliness causally affects future health problems. The health implications of loneliness outlined in this review, some of which are irreversible and can induce suffering and impairment, underscore the importance of interventions aimed at alleviating loneliness as a preventive strategy against both mental and physical illnesses.

## Einleitung

Einsamkeit gewinnt in der modernen Gesellschaft immer mehr an Bedeutung und wird zunehmend als ernstzunehmendes Gesundheitsrisiko auch außerhalb der Wissenschaft anerkannt. Dabei ist die Erkenntnis, dass Einsamkeit ein Gesundheitsrisiko darstellt, nicht neu. Bereits Ende der 1980er-Jahre wurden erste Übersichtsarbeiten zu den gesundheitlichen Konsequenzen von Einsamkeit veröffentlicht [1]. Dennoch hat es Einsamkeit erst im Laufe der letzten Dekade – speziell auch nach Bekanntwerden der Anstiege in der Einsamkeitsprävalenz während und nach der Covid-19-Pandemie [2] – in den gesellschaftlichen und gesundheitspolitischen Diskurs geschafft und differenzierte wissenschaftliche Erkenntnisse zu den Auswirkungen von Einsamkeit auf die psychische und körperliche Gesundheit sowie zu den verschiedenen Mechanismen, die diesen Zusammenhang bedingen, wurden gewonnen.

Die vorliegende narrative Übersichtsarbeit hat das Ziel, den breiten Wissensstand zu Einsamkeit als Gesundheitsrisiko zusammenzufassen und zu diskutieren. Basierend auf einer systematischen Literaturrecherche zu bestehenden Metaanalysen und systematischen sowie narrativen Reviews zu Einsamkeit als Gesundheitsrisiko beleuchtet dieser Artikel die vielfältigen Dimensionen von Einsamkeit und ihre gesundheitlichen Konsequenzen. Durch die Analyse bereits publizierter Übersichtsarbeiten und theoretischer Modelle wird ein Beitrag zum besseren Verständnis der Mechanismen geleistet, die Einsamkeit zu einem Risikofaktor für verschiedene psychische und körperliche Erkrankungen machen.

Einsamkeit wird definiert als die wahrgenommene Diskrepanz zwischen den bestehenden und den gewünschten sozialen Beziehungen [3]. Diese Diskrepanz kann sich sowohl hinsichtlich der Qualität als auch hinsichtlich der Quantität der sozialen Beziehungen zeigen. Zentral an dieser Definition ist zum einen, dass Einsamkeit eine subjektive Perspektive auf die sozialen Beziehungen beschreibt, die von objektiver sozialer Isolation abzugrenzen ist. Zum anderen verdeutlicht die Definition von Einsamkeit, dass sowohl die Qualität der sozialen Beziehungen (z. B. die empfundene emotionale Nähe in Beziehungen) als auch die Quantität (z. B. die Anzahl an Gesprächen pro Woche) eine Rolle spielen. In einer deutschen Repräsentativbefragung berichteten im Jahr 2021 etwa 42 %, dass sie sich zumindest manchmal einsam fühlen [4]. Angesichts der steigenden Prävalenz von Einsamkeit bei jungen Erwachsenen und mittelalten Erwachsenen [5, 6], ist es von entscheidender Bedeutung, die Zusammenhänge zwischen Einsamkeit und gesundheitlichen Beeinträchtigungen zu verstehen.

## Methodische Informationen

Dieser Artikel präsentiert zunächst die aus der systematischen Literaturrecherche identifizierten und für aussagekräftig erachteten Zusammenhänge zwischen Einsamkeit und psychischer sowie körperlicher Gesundheit. Anschließend werden die Mechanismen erörtert, durch die Einsamkeit Gesundheitseinschränkungen bewirken kann. Zudem werden methodische Herausforderungen bei der Erforschung von Einsamkeit als Gesundheitsrisiko beleuchtet. Ein abschließendes Fazit fasst die Kernaussagen zusammen.

Methodisches Vorgehen der systematischen Literaturrecherche:

- systematische Literaturrecherche in den Datenbanken PsycInfo und Medline (Januar 2024),
- Suchbegriffe in Titel und Abstract: „disorder, health, illness, morbidity, mortality, lonel*, meta-analysis, and review“ (nur englischsprachige Artikel),
- *k* = 756 Suchtreffer nach Entfernung von Duplikaten,
- zusätzliche relevante Artikel durch manuelle Suche ergänzt.

Kodierung der Suchtreffer:

- Abstracts und Titel wurden hinsichtlich der Relevanz von 4 unabhängigen Personen kodiert (Übereinstimmung > 80 %),
- *k* = 204 Metaanalysen und Reviews als thematisch passend eingestuft,
- bei Vorliegen mehrerer Metaanalysen und Reviews zu einem Thema wurden die umfangreichsten Beiträge berücksichtigt oder diejenigen, die neue Impulse lieferten (z. B. zu anderer Altersgruppe),
- Selektion der aussagekräftigsten Studien durch die Autorinnen für narratives Review.

## Einsamkeit und psychische Gesundheit

Einsamkeit zeigt insgesamt über alle Altersgruppen hinweg einen positiven Zusammenhang zu psychischen Störungen z. B. [7–9]. Caple et al. [7] identifizierten für Personen im Erwachsenenalter unter anderem finanzielle Belastungen, Arbeitslosigkeit und soziale Deprivation als situative Faktoren, die vermehrt bei Personen mit psychischen Störungen auftreten und mit dem Erleben von Einsamkeit einhergehen können. Einsamkeit sagt außerdem das Auftreten und die Schwere der psychischen Symptome vorher und sie kann die Erholung von einer psychischen Störung erschweren [7]. Eine geringe Größe des sozialen Netzwerks und eine schlechte soziale Integration sind weitere Faktoren, die häufig mit dem Erleben von Einsamkeit einhergehen und die Erholung von einer psychischen Störung erschweren [7]. Des Weiteren spielen psychologische Faktoren wie Gefühle von Wertlosigkeit, Hoffnungslosigkeit und fehlender Zugehörigkeit eine Rolle bei psychischen Störungen und können mit Einsamkeit zusammenhängen [7]. Im Folgenden werden die Ergebnisse ausgewählter Übersichtsarbeiten zum Zusammenhang von Einsamkeit und einzelnen psychischen Störungsbildern zusammengefasst.

### Depression und Suizidalität

Eine der am häufigsten untersuchten gesundheitlichen Folgen von Einsamkeit innerhalb der psychischen Störungsbilder ist die Depression. Metaanalysen und Literaturreviews finden über die gesamte Lebensspanne hinweg einen positiven Zusammenhang zwischen Einsamkeit und dem späteren Auftreten einer Depression [10, 11]. Dieser Zusammenhang erwies sich als konsistenter für die subjektiv empfundene Einsamkeit als für die objektive soziale Isolation [11]. Ein Literaturreview zeigte, dass die Depression im Kindes- und Jugendalter von allen untersuchten psychischen Störungen die stärkste Assoziation zur Einsamkeit aufweist [8]. Einsamkeit konnte Probleme der psychischen Gesundheit bis zu 9 Jahre im Voraus vorhersagen, wobei einige Befunde darauf hinweisen, dass die Dauer der erlebten Einsamkeit einen Effekt auf diesen Zusammenhang hat [8]. Mann et al. [10] fanden heraus, dass nach Kontrolle für konfundierende Variablen, wie z. B. soziale Unterstützung, einsame Personen eine mehr als doppelt so hohe Wahrscheinlichkeit aufweisen, im späteren Verlauf eine Depression zu entwickeln, wie nicht einsame Personen. Einige Studien weisen darauf hin, dass der Zusammenhang zwischen Einsamkeit und Depression für Mädchen stärker ist als für Jungen [8].

Des Weiteren zeigen aktuelle Forschungsbefunde, dass Einsamkeit in allen Altersgruppen mit der Symptomschwere einer Depression mehrere Monate bis Jahre später zusammenhängt [8, 12–14]. Höhere Einsamkeit sagte in diesen Literaturreviews die Verstärkung der bestehenden Depressionssymptomatik vorher. Dieser Effekt blieb in den meisten Studien bestehen, wenn für die Depressionssymptome zum ersten Messzeitpunkt kontrolliert wurde (z. B. [13]). Personen im Erwachsenenalter, die eine Depression aufwiesen, tendierten allerdings auch dazu, ihre soziale Unterstützung als inadäquat zu bewerten und sich einsam zu fühlen [13], weshalb es sich hierbei um einen bidirektionalen Zusammenhang handeln könnte. Einsamkeit erhöht die Wahrscheinlichkeit der Entwicklung einer Depression (z. B. [8, 10, 11]). Sozialer Rückzug infolge einer Depression und das Verschweigen der eigenen Probleme gegenüber dem sozialen Umfeld können wiederum die empfundene Einsamkeit erhöhen [15] und somit die Depressionssymptome verstärken. Literaturreviews sowohl querschnittlicher als auch längsschnittlicher Studien bestätigen, dass Einsamkeit in allen Altersgruppen die Wahrscheinlichkeit einer Remission der Depression verringert [7, 12, 13]. Dieser Effekt wurde für viele psychische Störungsbilder gefunden und innerhalb der Depression vor allem auf den Aspekt der internalisierten Stigmatisierung bezogen, die bei depressiven Patient*innen auftreten [7]. Diese Stigmatisierungen gehen mit Einsamkeit und einem Gefühl von Wertlosigkeit einher und verringern den Selbstwert und die Selbstwirksamkeit betroffener Personen. So kommt es zu einer Veränderung der individuellen Wahrnehmung der sozialen Beziehungen, was die Genesung von Depression erschwert [7].

Ein Literaturreview zu selbstverletzendem Verhalten im hohen Lebensalter zeigte außerdem, dass Einsamkeit positiv mit selbstverletzendem Verhalten zusammenhängt und teilweise als Motivation hierfür angegeben wird [16]. Von den Personen, die selbstverletzendes Verhalten zeigen, weisen knapp 70 % ebenfalls eine Depression auf [16]. Neben selbstverletzendem Verhalten steht auch Suizidalität über alle Altersgruppen hinweg mit Einsamkeit in positivem Zusammenhang [17–19]. Metaanalysen längsschnittlicher Studien zeigen, dass Einsamkeit einen Prädiktor für Suizidgedanken, -versuche und Suizid darstellt [20, 21]. In der Metaanalyse von Chang et al. [20] wiesen einsame Personen ein mehr als doppelt so hohes Risiko für Suizidalität auf als nicht einsame Personen. Ein möglicher vermittelnder Prozess in diesem Zusammenhang könnte eine bestehende Depression sein, da diese als Mediator zwischen Einsamkeit und Suizidalität identifiziert wurde [21]. Der Zusammenhang zwischen Einsamkeit und Suizidalität zeigte sich insbesondere für Altersgruppen von 16 bis 20 Jahren und über 58 Jahren sowie für weibliche Personen [21]. Es ist jedoch anzumerken, dass Personen im mittleren Alter sowie Männer in den betrachteten Studien unterrepräsentiert waren, was es erschwert, einen möglicherweise bestehenden (kleinen) Effekt in diesen Gruppen zu finden.

### Angststörungen

Unter den Angststörungen zeigt die soziale Phobie im Kindes- und Jugendalter den stärksten Zusammenhang zur Einsamkeit [8]. Einige Studien deuten darauf hin, dass dieser Zusammenhang für Jungen stärker ist als für Mädchen [8]. Literaturreviews belegen auch für das Erwachsenenalter, dass Einsamkeit sowie soziale Isolation mit dem Auftreten einer sozialen Phobie zusammenhängen [9, 22–24].

Längsschnittliche Studien mit Teilnehmenden unterschiedlicher Altersgruppen fanden überwiegend signifikante verstärkende Effekte von Einsamkeit auf Angststörungen, insbesondere die soziale Phobie [8, 11]. Die Auswirkungen von Einsamkeit auf andere Angststörungen sind im Vergleich dazu größtenteils unerforscht [9]. Studien, die den Zusammenhang von Einsamkeit und allgemeinen Angstsymptomen untersuchten, fanden für alle Altersgruppen positive Zusammenhänge [11].

### Abhängigkeitserkrankungen

Befunde aus einem Literaturreview deuten darauf hin, dass Einsamkeit im Jugend- und Erwachsenenalter ebenfalls mit Abhängigkeitserkrankungen in Zusammenhang steht [25]. Schwerwiegendere und länger bestehende Abhängigkeitserkrankungen gingen in diesem Literaturreview mit stärkerer Einsamkeit einher. Es wurde gezeigt, wie auch in einzelnen anderen längsschnittlichen Studien, dass Einsamkeit einen Risikofaktor für das Fortbestehen der Abhängigkeitserkrankung darstellt und dass der Substanzgebrauch oft zur Bewältigung negativer Emotionen wie Einsamkeit eingesetzt wird. Insgesamt konnten jedoch keine konsistenten Befunde bezüglich der kausalen Richtung des Zusammenhangs gefunden werden.

Weitere Literaturreviews beschäftigen sich mit Einsamkeit im Kontext spezifischer Abhängigkeitserkrankungen. Einsamkeit stellt beispielsweise einen Prädiktor für den Beginn von problematischem Alkoholkonsum im höheren Lebensalter dar [26]. In Bezug auf das Rauchen und exzessives Glücksspielverhalten im Erwachsenenalter gibt es uneinheitliche Befunde [27, 28]. Einige Studien fanden positive Zusammenhänge zwischen Einsamkeit und diesen Verhaltensweisen, während andere keine Zusammenhänge aufdecken konnten [27, 28]. Die Uneinheitlichkeit in den Ergebnissen könnte teilweise auf eine geringe Qualität einiger eingeschlossenen Studien zurückzuführen sein [27]. Es gibt Hinweise darauf, dass einige Personen mit exzessivem Glücksspielverhalten durch die sozialen Kontakte während des Glücksspiels versuchen, Einsamkeitsgefühle zu verringern, was zur Aufrechterhaltung dieses Verhaltens beitragen könnte [28]. In einer aktuellen Metaanalyse die sich unter anderem mit Internet-Spielsucht bei Jugendlichen beschäftigt, wurde Einsamkeit ebenfalls als Risikofaktor für die Entwicklung einer Internet-Spielsucht identifiziert [29]. Als eine mögliche Erklärung wird hier ebenfalls die Suche nach sozialen Kontakten durch Internetspiele angeführt. Es ist jedoch anzumerken, dass innerhalb dieser Metaanalyse nur 2 Studien Einsamkeit erfassten, daher sollten die Ergebnisse vorsichtig interpretiert werden.

### Abbau kognitiver Funktionen

Befunde aus Literaturreviews zeigen, dass Einsamkeit im mittleren und späten Erwachsenenalter sowohl in Industrieländern als auch in Ländern mit niedrigen und mittleren Einkommen mit kognitiven Beeinträchtigungen und einer Verschlechterung kognitiver Funktionen zusammenhängt [30–32]. Auch längsschnittliche Studien zeigen, dass Einsamkeit einen Risikofaktor für die Verschlechterung kognitiver Funktionen im hohen Lebensalter darstellt [22]. Einige Studien deuten auf einen bidirektionalen Zusammenhang zwischen diesen Konstrukten hin [30].

Es gibt robuste Befunde, die zeigen, dass Einsamkeit im hohen Lebensalter einen Risikofaktor für die Entwicklung dementieller Erkrankungen darstellt [19, 22, 33]. Tragantzopoulou und Giannouli [9] zeigten, dass einsame Personen im hohen Lebensalter eine 2‑mal so hohe Wahrscheinlichkeit haben, an der Alzheimer-Krankheit zu erkranken, wie nicht einsame Personen. Über einen Zeitraum von 15 Jahren erwies sich die Qualität sozialer Kontakte (z. B. Zufriedenheit durch soziale Interaktionen) als protektiver Faktor für die Alzheimer-Krankheit während die Quantität (z. B. Größe des sozialen Netzwerks) keinen Einfluss hatte [9].

### Weitere psychische Störungen

Befunde aus Literaturreviews zeigen, dass Einsamkeit im Erwachsenenalter mit Schlafproblemen und Schlafstörungen zusammenhängt [34–36]. Bislang gibt es nur wenig Forschung zu Langzeitfolgen von Einsamkeit auf den Schlaf. In einzelnen längsschnittlichen Studien sagte Einsamkeit die Schlafqualität oder Schlafzufriedenheit zu einem späteren Messzeitpunkt vorher, andere fanden hingegen keinen Effekt [35]. Objektive Messungen deuten darauf hin, dass einsame Personen sowohl im frühen als auch im späten Erwachsenenalter keine geringere Schlafdauer, aber eine geringere Schlafqualität aufweisen, als nicht einsame Personen [37]. Da Schlafstörungen oft komorbid mit Angststörungen und affektiven Störungen auftreten, könnte der Zusammenhang zwischen Schlafstörungen und Einsamkeit auch über diese anderen Störungsbilder vermittelt sein.

Ein Literaturreview zu Einsamkeit und Persönlichkeitsstörungen zeigte, dass Personen mit Persönlichkeitsstörungen und Personen mit höheren Ausprägungen maladaptiver Persönlichkeitseigenschaften (ausgenommen narzisstischen Eigenschaften) einsamer sind als die allgemeine Bevölkerung oder andere klinische Stichproben [38]. Einzelne längsschnittliche Befunde deuten auch darauf hin, dass Einsamkeit über die Zeit hinweg positiv mit der Anzahl an maladaptiven Persönlichkeitseigenschaften zusammenhängt, die eine Person aufweist [38]. Aufgrund begrenzter Forschung in diesem Bereich und teilweise geringer Qualität der Studien sollten diese Befunde allerdings mit Vorsicht interpretiert werden [38].

Der Zusammenhang zwischen Einsamkeit und psychotischen Störungen ist größtenteils noch unerforscht [9, 39]. Bisherige Forschungsbefunde zum Erwachsenenalter geben Hinweise darauf, dass Personen mit psychotischen Störungen einsamer sind als Personen aus der allgemeinen Bevölkerung und dass Einsamkeit und soziale Isolation Risikofaktoren für die Entwicklung einer Psychose darstellen [9, 39]. Positive Zusammenhänge mit Einsamkeit wurden außerdem sowohl für das Auftreten von Positiv- und Negativsymptomen psychotischer Störungen gefunden als auch für die Symptomschwere der Psychose im Erwachsenenalter [11]. Einsamkeit weist im Kindes- und Jugendalter zudem einen positiven Zusammenhang mit der Aufmerksamkeitsdefizit-Hyperaktivitätsstörung und der Autismus-Spektrum-Störung auf [12, 40].

## Einsamkeit und körperliche Gesundheit

Über verschiedene Messansätze und Studien hinweg deutet die empirische Evidenz darauf hin, dass sozial besser vernetzte Menschen gesünder sind und länger leben (z. B. [41–43]). Besonders hervorzuheben ist hier epidemiologische und psychologische Forschung, die die Frage zum Zusammenhang zwischen Einsamkeit und Gesundheit prospektiv in großen Stichproben untersucht, indem sie die soziale Vernetzung und Einsamkeit von Individuen gemessen und die Entwicklung dieser Personen dann über die Zeit, oft über Jahrzehnte, hinweg weiterverfolgt hat. Auf diese Weise konnte bestimmt werden, ob soziale Verbundenheit bzw. Einsamkeit bestimmte körperliche Erkrankungen, das Überleben oder die Zeitspanne bis zum Tod vorhersagt. Die folgenden Abschnitte beschreiben mehrere aktuelle Übersichtsarbeiten und Metaanalysen, die die relevanten Daten zu diesen Fragen zusammenfassen.

### Herz-Kreislauf-Erkrankungen

Verschiedene Übersichtsarbeiten berichten, dass vor allem Herz-Kreislauf-Erkrankungen im Zusammenhang mit Einsamkeit und objektiver sozialer Isolation untersucht wurden (z. B. [44–46]). Diese Metaanalysen und Literaturreviews zeigen basierend auf Längsschnittdaten, dass Erwachsene, die sich chronisch einsam fühlen, ein erhöhtes Risiko haben, zu einem späteren Zeitpunkt an einer Herz-Kreislauf-Erkrankung zu leiden.

Im Jahr 2020 berichtete zudem ein Unterausschuss der Nationalen Akademien der Wissenschaften und Medizin aus den USA, dass es einen Anstieg des Risikos für Herz-Kreislauf-Erkrankungen um ein Drittel bei Personen mit schlechter sozialer Gesundheit, insbesondere sozialer Isolation und Einsamkeit, gegeben hat [47]. Dieser Befund basierte auf einer systematischen Übersichtsarbeit und Metaanalyse, in der gezeigt wurde, dass Einsamkeit und soziale Isolation das Risiko für das Auftreten von koronarer Herzkrankheit um 29 % und für Schlaganfall um 32 % erhöhen [48]. Diese Ergebnisse unterschieden sich nicht signifikant in Hinblick auf das Geschlecht und andere Merkmale. Konträr zu diesen Ergebnissen berichtet eine andere Metaanalyse keinen Zusammenhang zwischen Einsamkeit und selbstberichteten Herz-Kreislauf-Erkrankungen [49]. Eine Einschränkung dieser Metaanalyse liegt jedoch in der Abhängigkeit von selbstberichteten Feststellungen von Herz-Kreislauf-Erkrankungen in den eingeschlossenen Längsschnittstudien.

In verschiedenen systematischen Literaturreviews zeigen sich Hinweise darauf, dass der gefundene robuste Zusammenhang von Einsamkeit mit objektiver sozialer Isolation und Mortalität über das Herz-Kreislauf-System und die psychische Gesundheit vermittelt wird (für eine Übersicht siehe [50]). Dieser Zusammenhang scheint sowohl für den subjektiven Zustand der Einsamkeit als auch für den objektiven Zustand der sozialen Isolation zuzutreffen, aber die konsistentesten signifikanten Effekte wurden in Bezug auf Messungen der sozialen Isolation berichtet [50].

Soziale Isolation scheint zudem nicht nur mit dem Auftreten einer Herz-Kreislauf-Erkrankung, sondern auch mit der Erholung von Herz-Kreislauf-Erkrankungen verbunden zu sein. Schlaganfall-Patient*innen mit einem hohen Niveau an sozialer Unterstützung oder großen sozialen Netzwerken zeigten eine schnellere und umfassendere funktionelle Erholung nach einem Schlaganfall im Vergleich zu sozial isolierten Personen [51, 52]. Für Einsamkeit gibt es diesbezüglich wenig empirische Evidenz.

### Mortalität

Mehrere Übersichtsarbeiten wurden identifiziert, die den Zusammenhang zwischen Einsamkeit und Mortalität untersuchen. Eine Metaanalyse, die den Zusammenhang zwischen Mortalität und sowohl Einsamkeit als auch sozialer Isolation und Alleinleben bei Personen aus der Allgemeinbevölkerung untersucht, zeigte, dass alle 3 Indikatoren für fehlende soziale Beziehungen das Mortalitätsrisiko signifikant erhöhten [41]. Die gewichteten durchschnittlichen Effektgrößen zeigten eine Erhöhung der Sterbewahrscheinlichkeit um jeweils 29 % (für soziale Isolation), 26 % (für Einsamkeit) und 32 % (für Alleinleben). Die Ergebnisse unterschieden sich nach dem Alter der Teilnehmenden, wobei soziale Defizite bei Personen mit einem Durchschnittsalter von unter 65 Jahren stärker prädiktiv für die Mortalität waren.

Eine weitere aktuelle Metaanalyse mit Personen aus der Allgemeinbevölkerung ([53], die ein Update der Ergebnisse von Holt-Lunstadt et al. [41] darstellt) bestätigte, dass sowohl soziale Isolation als auch Einsamkeit signifikant mit einem erhöhten Risiko für die Sterblichkeit unabhängig von den genauen Ursachen verbunden war. Ebenfalls die Allgemeinbevölkerung untersuchend bestätigte eine weitere Metaanalyse [54] Einsamkeit als einen Risikofaktor für Mortalität. Die jüngste Metaanalyse [55] zum Zusammenhang zwischen Einsamkeit und Mortalität bei älteren Erwachsenen ergab, dass sowohl Einsamkeit als auch eine geringe Größe des sozialen Netzwerks mit einem erhöhten Sterberisiko bei älteren Erwachsenen verbunden sind.

### Gebrechlichkeit

Mehrere Metaanalysen und Literaturreviews beschäftigen sich mit dem Zusammenhang zwischen Einsamkeit und Gesundheitseinschränkungen bei hochaltrigen Menschen (z. B. [42]). In diesem Kontext wird vor allem der Zusammenhang zwischen Einsamkeit und Gebrechlichkeit beschrieben. Gebrechlichkeit ist ein mehrdimensionales Syndrom, das sich durch abnehmende Funktionsreserven und geringere Widerstandsfähigkeit gegen Stress äußert und die körperliche Leistungsfähigkeit, Ernährung, psychische Gesundheit und Kognition beeinträchtigen kann [56]. In einer Übersichtsarbeit [57] offenbarten die meisten der einbezogenen Studien (Stichproben aus im häuslichen Umfeld lebenden älteren Menschen) Zusammenhänge zwischen sozialer Isolation, Einsamkeit und Gebrechlichkeit. Der Großteil der Studien fand Belege für Assoziationen zwischen Gebrechlichkeit und negativen sozialen Auswirkungen; jedoch zeigten nur wenige Studien eine Beziehung zwischen sozialer Isolation und Gesundheitseinschränkungen. Trotz der etablierten Verbindung zwischen Gebrechlichkeit und negativen sozialen Auswirkungen untersuchte laut den Autor*innen keine Studie, *auf welche Weise* soziale Isolation und Einsamkeit die negativen Folgen von Gebrechlichkeit verursachen können [57].

### Weitere körperliche Erkrankungen

Vereinzelt wurden zudem Metaanalysen und Literaturreviews zu anderen körperlichen Erkrankungen identifiziert, die im Folgenden präsentiert werden. Eine Metaanalyse berichtete einen Zusammenhang zwischen schlechterer Mundgesundheit und Einsamkeit sowie sozialer Isolation [58]. In diese Metaanalyse sind überwiegend Querschnittstudien eingeflossen und die Kausalbeziehung zwischen Einsamkeit und Zahngesundheit ist unklar.

Unter Verwendung des *Global School-based Student Health Survey* (87.269 Personen aus 26 Ländern) wurde der Zusammenhang zwischen psychosozialen Umständen und Verletzungen bei Jugendlichen untersucht [59]. Ein starker positiver Zusammenhang bestand zwischen schweren Verletzungen und Einsamkeit.

Im Rahmen unserer systematischen Literaturrecherche konnte kein Review und keine Metaanalyse identifiziert werden, die Einsamkeit im Zusammenhang mit Diabetes untersucht. Jedoch zeigte eine kürzlich veröffentlichte große Längsschnittstudie, dass Einsamkeit das Risiko für eine spätere Typ-2-Diabetes-Erkrankung deutlich erhöhte [60]. Teilnehmende, die bei der ersten Befragungswelle in den Jahren 1995–1997 „sehr starke“ Einsamkeit angaben, hatten 20 Jahre später in der Befragungswelle in den Jahren 2017–2019 eine doppelt so hohe Wahrscheinlichkeit, an Typ-2-Diabetes erkrankt zu sein, im Vergleich zu denen, die sich nicht einsam fühlten.

## Mechanismen: Wie führt Einsamkeit zu Gesundheitseinschränkungen?

Einsamkeit und soziale Isolation haben signifikante Auswirkungen auf gesundheitsbezogenes Verhalten und führen zu verstärkten autonomen Stressreaktionen sowie einer Hyperaktivität des sympathischen Nervensystems. Beispielsweise wird die schlechte Einhaltung und Befolgung einer vorgegebenen Behandlung bei von Einsamkeit betroffenen oder sozial isolierten Menschen [61] als Mechanismus für den Zusammenhang zwischen Einsamkeit und Gesundheitseinschränkungen diskutiert. Auch zeigen Metaanalysen und Literaturreviews Zusammenhänge zwischen Einsamkeit und geringerer körperlicher Aktivität [62, 63], die auch in Längsschnittstudien gefunden wurden, bei denen Einsamkeit zu einem Messzeitpunkt geringere körperliche Aktivität zu einem späteren Messzeitpunkt vorhersagte [64]. Diese geringe körperliche Aktivität wird als ein Mechanismus diskutiert, der von Einsamkeit zu Gesundheitseinschränkungen führt [65].

Für den Zusammenhang zwischen Einsamkeit und Herz-Kreislauf-Erkrankungen wird argumentiert, dass die verstärkten autonomen Stressreaktionen und die Hyperaktivität des sympathischen Nervensystems mit einer Zunahme des totalen peripheren Widerstands (TPR) und einer Verringerung der Variabilität der Herzfrequenz (HRV) assoziiert sind, was wiederum zu Hypertonie, ischämischer Herzkrankheit und verminderter Herzleistung beiträgt [66]. Tierexperimentelle Studien zeigen, dass soziale Isolation mit erhöhten Basalwerten von Glukokortikoiden korreliert, die durch eine Überstimulation der Hypothalamus-Hypophysen-Nebennierenrinden-Achse verursacht werden. Dies führt zu chronischer Entzündung und Glukokortikoidresistenz, gekennzeichnet durch eine Rezeptordesensibilisierung. Ähnliche Mechanismen werden auch in weiteren Übersichtsarbeiten beschrieben [67].

## Methodische Herausforderungen bei der Untersuchung von Einsamkeit als Gesundheitsrisiko

Die Untersuchung von Einsamkeit als Gesundheitsrisiko stellt Forschende vor eine Reihe methodischer Herausforderungen, insbesondere wenn es darum geht, die kausalen Effekte von Einsamkeit zu einem Zeitpunkt auf die Gesundheit zu einem späteren Zeitpunkt zu schätzen. Eine zentrale Herausforderung liegt in der Notwendigkeit, Längsschnittstudien durchzuführen, die es ermöglichen, Veränderungen im Gesundheitszustand der Teilnehmenden über die Zeit zu beobachten und dabei das ursprüngliche Gesundheitsniveau sowie andere konfundierende Variablen zu kontrollieren. Solche Variablen können sowohl die unabhängige Variable, in diesem Fall die Einsamkeit, als auch die abhängige Variable, also die Gesundheit, beeinflussen und müssen daher sorgfältig berücksichtigt werden, um valide Schlüsse über kausale Beziehungen ziehen zu können. Hinzu kommt, dass Einsamkeit und Gesundheitszustände wechselseitig aufeinander einwirken können, was die Bestimmung der Kausalrichtung erschwert. Beispielsweise kann chronische Einsamkeit zu Gesundheitsproblemen führen, während gleichzeitig bestehende Gesundheitsprobleme ein Individuum anfälliger für Einsamkeit machen können. Tatsächlich zeigte sich beispielsweise für Depression [68] und soziale Angst [69], dass diese Einsamkeit bedingen und von Einsamkeit beeinflusst werden, sodass hier ein Teufelskreis entstehen kann.

Um diese Herausforderungen zu bewältigen, ist es entscheidend, in Längsschnittstudien statistische Methoden anzuwenden, die es erlauben, für das Ausgangsniveau der Gesundheit und für konfundierende Variablen zu kontrollieren. Dazu gehören multivariate Analysemethoden, die gleichzeitig den Einfluss mehrerer unabhängiger Variablen auf eine abhängige Variable untersuchen können.

Die kritische Betrachtung der in dieser Übersichtsarbeit zusammengefassten Metaanalysen und Reviews zeigt, dass viele Studien zum Zusammenhang zwischen Einsamkeit und Gesundheit querschnittliche Designs verwenden und aufgrund fehlender Kontrolle von konfundierenden Variablen keine Kausalschlüsse zulassen. Zukünftige Forschung, die sich mit Einsamkeit als Gesundheitsrisiko beschäftigt, sollte so gestaltet sein, dass neben den Kausalzusammenhängen idealerweise auch die Mechanismen für den Zusammenhang beleuchtet werden.

## Fazit

Vielfältige Forschungsarbeiten legen eine enge Verknüpfung von Einsamkeit und Einbußen in der psychischen und körperlichen Gesundheit nahe. Diese Erkenntnis impliziert, dass eine wirksame, idealerweise evidenzbasierte Einsamkeitsintervention (für Anregungen s. [70]) auch präventiv gegen die Entstehung von psychischen und körperlichen Erkrankungen wirken kann. Um Einsamkeit als Gesundheitsrisiko jedoch noch besser zu verstehen, sind hochauflösende Längsschnittstudien notwendig, die idealerweise auch eine Untersuchung der verhaltensbezogenen und (neuro)biologischen Mechanismen erlauben.
